# Cold Spot SCANNER: Colab Notebook for predicting cold spots in protein–protein interfaces

**DOI:** 10.1186/s12859-024-05796-5

**Published:** 2024-04-30

**Authors:** Sagara N. S. Gurusinghe, Julia M. Shifman

**Affiliations:** Department of Biological Chemistry, The Alexander Silberman Institute of Life Sciences, The Hebrew University of Jerusalem, Jerusalem, Israel

**Keywords:** Protein–protein interactions, Cold spots, Cavities, Binding interface, Binding affinity

## Abstract

**Background:**

Protein–protein interactions (PPIs) are conveyed through binding interfaces or surface patches on proteins that become buried upon binding. Structural and biophysical analysis of many protein–protein interfaces revealed certain unique features of these surfaces that determine the energetics of interactions and play a critical role in protein evolution. One of the significant aspects of binding interfaces is the presence of binding hot spots, where mutations are highly deleterious for binding. Conversely, binding cold spots are positions occupied by suboptimal amino acids and several mutations in such positions could lead to affinity enhancement. While there are many software programs for identification of hot spot positions, there is currently a lack of software for cold spot detection.

**Results:**

In this paper, we present Cold Spot SCANNER, a Colab Notebook, which scans a PPI binding interface and identifies cold spots resulting from cavities, unfavorable charge-charge, and unfavorable charge-hydrophobic interactions. The software offers a Py3DMOL-based interface that allows users to visualize cold spots in the context of the protein structure and generates a zip file containing the results for easy download.

**Conclusions:**

Cold spot identification is of great importance to protein engineering studies and provides a useful insight into protein evolution. Cold Spot SCANNER is open to all users without login requirements and can be accessible at: https://colab.research.google.com/github/sagagugit/Cold-Spot-Scanner/blob/main/Cold_Spot_Scanner.ipynb.

**Supplementary Information:**

The online version contains supplementary material available at 10.1186/s12859-024-05796-5.

## Background

Protein–protein interactions (PPIs) are physical associations between two or more proteins that play a crucial role in many biological functions [1]. PPIs are highly specific and tightly regulated, and any disruption of their function can result in a wide range of diseases [2]. Thus, developing inhibitors and activators of PPIs is a promising therapeutic approach [3]. Each PPI is characterized by a particular binding affinity, which is largely determined by intermolecular interactions at the PPI binding interface, i. e. the surface patch that becomes buried upon protein binding [4]. Binding interfaces contain several important residues called binding hot spots that contribute most significantly to the binding free energy and could not be mutated without considerable loss in binding affinity [5–9]. In contrast, cold spots are positions in the binding interface that are occupied by suboptimal amino acids; at such positions, mutations to several amino acids lead to binding affinity enhancement. These suboptimal amino acid positions are important in protein evolution as such positions can support formation of low-affinity and transient PPIs and convey multispecific interactions, which are not optimized for any particular partner protein. In our previous work, we identified three scenarios of how cold spots could occur [10]. In the first scenario, we observed that a wild-type residue in the binding interface does not interact with the binding partner, creating a cavity within the binding interface. Mutating such a residue to larger amino acids produces new intermolecular interactions and results in binding affinity enhancement. In the second scenario, a charged residue in the binding interface is buried in a hydrophobic environment, resulting in an unfavorable or frustrated interaction. Mutating such a residue to a non-charged residue eliminates unfavorable charged-hydrophobic interactions, enhancing binding affinity. In a third scenario, two amino acids of the same charge are found within close proximity of each other. Eliminating these unfavorable same-charge interactions through a mutation results in affinity improvement.

Previously, cold spots have been identified experimentally by performing deep mutational scanning of a protein and subsequent sorting of mutants for binding affinity with yeast surface or phage display technologies [11–13]. Such an approach provides a large amount of invaluable data but is labor-intensive. An alternative method for identifying cold spots is through computational approaches that predict changes in free energy of binding (ΔΔG_bind_) due to all possible mutations and subsequently identify cold spot positions that contain at least three affinity-enhancing mutations [14, 15]. While computational approaches are faster and more cost-effective than experimental methods, they still require lengthy calculations of ΔΔG_bind_ values and produce inaccurate results for at least some mutations. Computational methods that utilize physical atomic-based energy functions achieve correlation of 0.3–0.5 [16, 17] with experimental data while machine-learning-based approaches produce a higher correlation of 0.6–0.8 [18–20]. However, the latter heavily depend on previous experimental data of ΔΔG_bind_ and such data is frequently inconsistent, collected at different experimental conditions on different complexes and using different methods.

Recently, we introduced a new fast and highly efficient computational approach that identifies cold spots directly from protein structure and requires no experimental data to learn on. This method searches for unfavorable interactions inside protein–protein interfaces that include cavities, charge-hydrophobic, and same-charge interactions [21]. Using this protocol, we performed the analysis of nearly four thousand homo- and heterodimers in the PDB. We observed that cold spots due to cavities are present in nearly all PPIs unrelated to their binding affinity, while unfavorable charge-hydrophobic and same-charge interactions are not as frequent. We also found that most cold spots are located in the periphery of the binding interface, with high-affinity complexes showing fewer centrally located colds spots than low-affinity complexes. Furthermore, the analysis revealed that cold spots are more frequent in homo-dimeric complexes compared to hetero-complexes, likely due to symmetry constraints imposed on sequences of homodimers. Here, we introduce Cold Spot SCANNER, a Colab notebook that is fast and provides a convenient interface for identifying cold spots in any PPI using an experimentally determined structure or a structural model of the PPI as an input. The output provides an interactive 3D visualization of the protein complex showing cold spots in the binding interface on top of the PPI structure. The calculation also generates a result file for downloading. Cold Spot SCANNER could be used to analyze any protein–protein complex, providing information on the binding interface optimality and identifying positions that could be further engineered to enhance PPI binding affinity and/or specificity.

## Implementation

### Input files

**Fig. 1:**
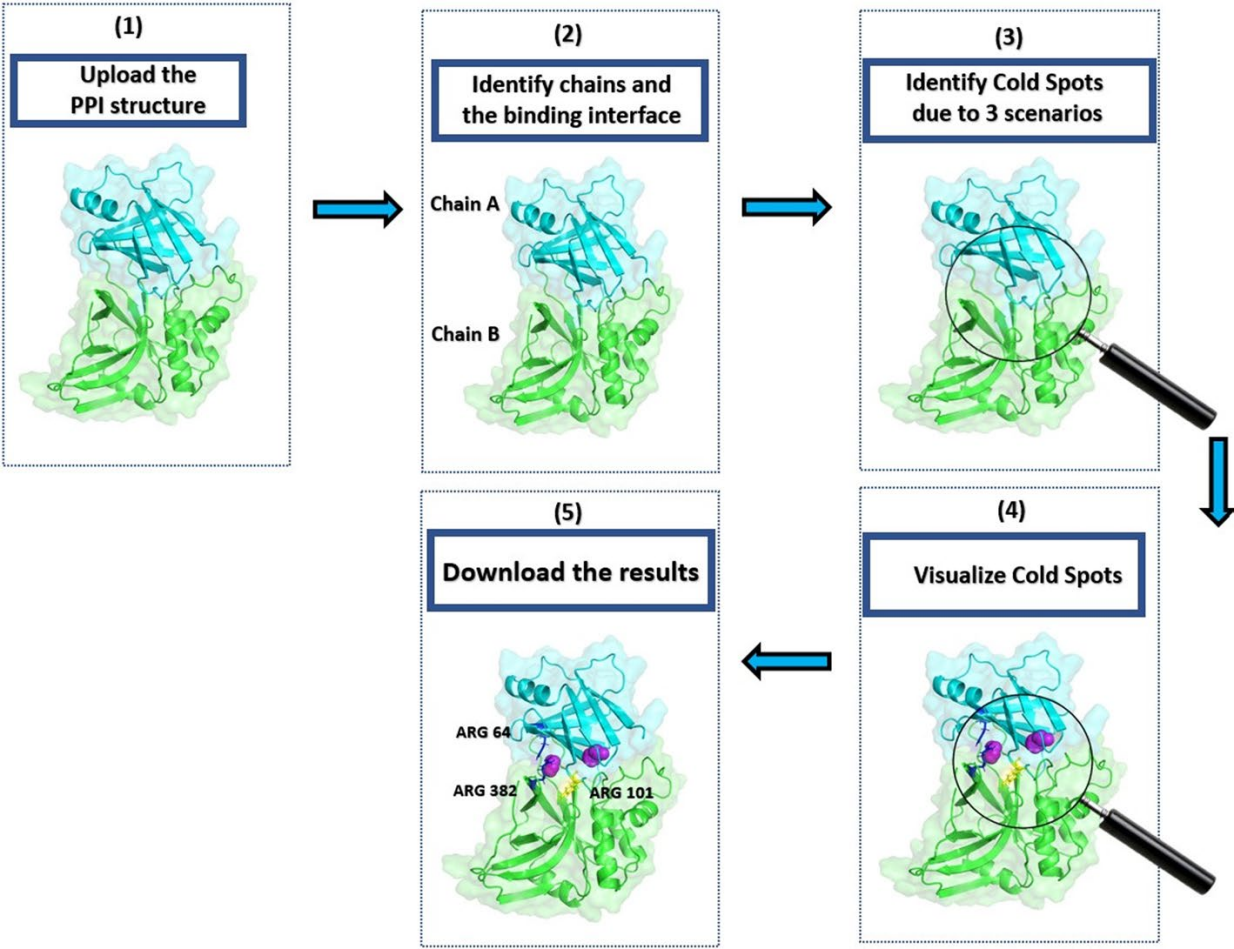
General Flow Chart for Cold Spot SCANNER. Identified cold spots in the complex between a cysteine protease and its inhibitor (PDB ID 1PXV). Two cold spots due to cavities (magenta), one cold spot due to charge-hydrophobic interaction (yellow), and two cold spots due to same-charge interactions (blue) were identified

Cold Spot SCANNER takes as an input a structure of a PPI complex, which could be either downloaded directly from the PDB or uploaded from the user’s computer. The PPI complex structure should contain at least two interacting chains. The user has to specify the chain identifiers and the program then computes the corresponding binding interface and identifies cold spots within this interface (Fig. 1). If wrong or non-interacting chains are given as input, an error will be generated. The input PDB file does not need to contain hydrogen atoms as hydrogens are added during the calculation. The user can use either X-ray crystallography or NMR structures as inputs for cold-spot predictions. If an NMR structure is given, the program will automatically extract the first model of the structure and use it for its predictions. If no experimentally available structure is available for a particular PPI, a modeled structure could be generated by AlphaFold-Multimer [22] and used as an input for Cold Spot SCANNER. Note that if a PPI complex structure contains heteroatoms within the binding interface, such heteroatoms will be ignored and a cold spot will be likely predicted due to a cavity. In addition, if some atoms are missing from amino acids belonging to the binding interface, a cold spot due to a cavity will be likely predicted in error. Hence, the input structures should be rebuilt to add the missing atoms prior to cold spot calculations.

### Cold spot identification

After the input file is submitted, Cold Spot SCANNER starts its predictions. Hydrogens are first added to the PDB file using the MolProbity software with asparagines, histidines and glutamines allowed to flip [23]. Then binding interface atoms are identified as all atoms on one chain that are within 4 Å from the second chain in the complex. The program then searches for cold spots in the binding interface occurring due to three scenarios as described below.

### Identification of cold spots due to unfavorable charge-hydrophobic interactions:

First, buried charged atoms in the binding interface are identified that are located within 4.5 Å from a hydrophobic atom on another amino acid. This cutoff was selected since it is in the middle of the range usually used to define direct residue-residue interaction [24, 25]. The software excludes the position from a cold spot count if this charged atom participates in any of the favorable interactions such as (1) hydrogen bonds, (2) pi-cation interactions, (3) favorable opposite charge interactions and (4) anion aromatic interactions as discussed in detail in [21]. If no exclusion rule is found, the charged atom is considered a cold spot due to charge-hydrophobic interactions.

### Identification of cold spots due to same-charge interactions

Charged atoms in the binding interface are identified that are located within 4.5 Å from the same charge belonging to a different amino acid. This cutoff was selected since it is in the middle of the range usually used to define direct residue-residue interaction [24, 25] similar to the procedure for charge-hydrophobic interactions. The software excludes charges that participate in hydrogen bonds or favorable opposite-charge interactions. If no exclusion rule is found, both charged residues in the unfavorable interaction are identified as cold spots.

#### Identification of cold spots due to cavities

To identify cold spots due to cavities, random dots are placed within the PPI binding interface. A surface is generated using a sphere of 2.4 Å rotated around the protein surface and the dots, which are outside of this surface are removed. This sphere radius was found to be optimal by varying the radius, identifying cavities and visually inspecting the structures using a large set of PPIs [21]. Inside the probe surface, the dots that are within the Van der Waals radius of any protein atom are removed and the rest of the dots are retained and represent the empty spaces or cavities. To cluster the dots into separate cavities, DBSCAN algorithm [26] is applied using the epsilon value set to 2 (maximum distance between the two points) and the minimum value set to 10 dots per cluster, which corresponds to a volume of a water molecule. Note, that if the PPI structure contains non-protein atoms in the binding interface, these atoms will be removed prior to the calculation and cavities will be computed in their place.

A calculation for a PPI with a medium size interface runs for 5–15 min.

## Results

Once the calculation is completed, the identified cold spots are visualized in an interactive structural picture generated using Py3DMOL. Cold spots due to cavities are shown as red spheres and cold spots due to charge-hydrophobic and same-charge interactions are shown as yellow and blue sticks, respectively. For a better view, the structure of the PPI could be rotated by clicking and turning the structure with the mouse. In addition, results will be automatically downloaded to the Download folder of the user computer as a zip file that contains three files: a csv file, which summarizes the identified cold spots (cavities, same-charge and charge-hydrophobic interactions), a .pdb file, which contains the 3D coordinates of the protein complex with the identified cavities and a PYMOL script file, which allows the user to visualize the PPI structure and all cold spots colored by type. An example of a calculation and its output is shown in the Supplementary Information (Additional file 1).

### Validating cold spot predictions

**Fig. 2:**
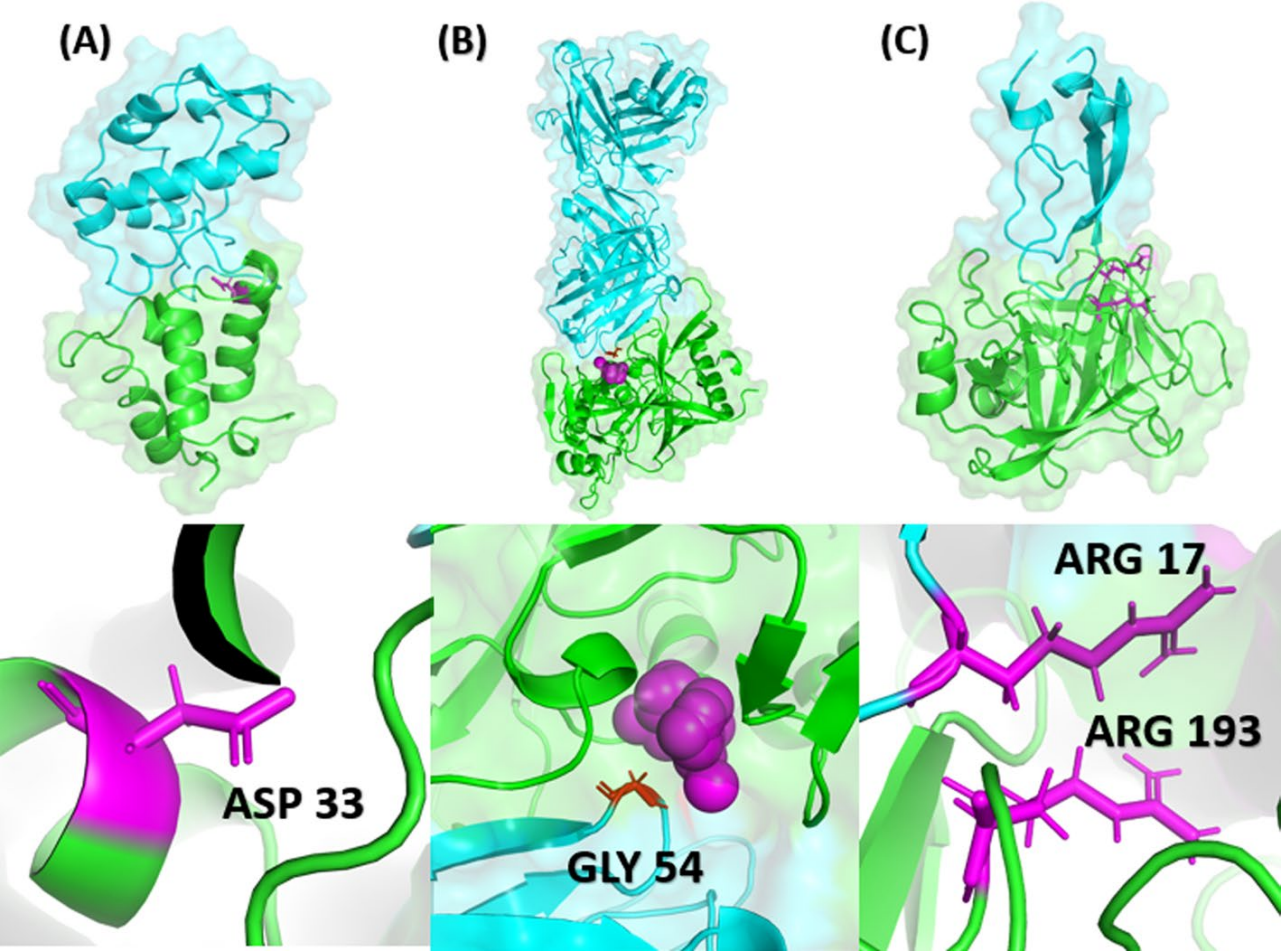
The top panel shows the identified cold spots in a specific PPI. The bottom panel zooms into the cold spots. Cold spots are shown in magenta. **A** A complex between Colicin E9 (cyan) and IM2 (green) (PDB 2WPT). **B** A complex between the HIV protein gp120 (green) and a broadly neutralizing HIV antibody (cyan) (PDB 3U7Y). **C** A complex between mesotrypsin (green) and BPTI (cyan) (PDB 2R9P)

No database of experimentally determined cold-spots exists at the moment. Hence, we assessed the validity of Cold Spot SCANNER by examining several exemplary PPIs where cold spots have been identified experimentally. Our first example is a complex between Colicin E9 and its non-cognate binding partner immunity protein 2 (IM2) (PDB 2WPT). Experimental measurements revealed that substituting the aspartate residue at position 33 in Im2 with a leucine led to a six orders of magnitude increase in binding affinity [17]. Cold Spot SCANNER identified position 33 as a cold spot due to an unfavorable charge-hydrophobic interaction (Fig. 2A). Our second example is the complex between the HIV protein gp120 and a broadly neutralizing HIV antibody (PDB 3U7Y). In this complex, replacing a glycine residue at position 54 on the antibody with aromatic residues improved binding affinity by three- to four-fold [18]. Cold Spot SCANNER identified a cavity adjacent to position 54 as a cold spot (Fig. 2B). Our third example is a complex between mesotrypsin and bovine pancreatic trypsin inhibitor BPTI (PDB 2R9P), where experiment demonstrated that mutating arginine at positions 17 on BPTI or 193 on mesotrypsin to several amino acids increased the PPI binding affinity [11]. Cold Spot SCANNER identified positions 17 and 193 as cold spots due to same charge interaction (Fig. 2C). The running time of Cold Spot SCANNER can vary depending on the size of the binding interface with an average calculation taking 10–15 min. While certain methods [27, 28] could predict changes in binding affinity due to mutations within seconds to minutes, these protocols require manual identification of the binding interface residues, manual specification of mutations and subsequent analysis of the produced data for cold spot assignment. Hence, our Cold Spot SCANNER stands as the only method for identification of cold spots seamlessly, without performing additional calculations and data processing.

## Conclusions

While many available webs servers exist for computing ΔΔG_bind_ due to single mutations, there is currently no server for direct prediction of cold spots of binding, the imperfections in PPI binding interfaces that are found frequently in complexes with various affinities and functions. To fill in this gap, we developed Cold Spot SCANNER for fast and accurate identification and analysis of cold spots in various PPIs. Cold spot identification is of great importance to protein engineering studies and provides a useful insight into protein evolution. Cold spot positions can be utilized by researchers to enhance PPI binding affinity, primarily through the introduction of mutations at the cold spot positions [29]. As the probability of finding affinity-enhancing mutations at cold spots is high, these positions should be the focus of randomization in protein engineering experiments that design protein therapeutics. In addition, cold spots identified as cavities can serve as potential targets for small molecules, with the goal to stabilize a particular PPI [30]. Cold Spot SCANNER provides researchers with a convenient and user-friendly means of identifying cold spots in PPIs and could greatly assist future studies of protein evolution and design.

### Availability and requirements

- *Project name* Cold Spot SCANNER.
- *Project home page* https://colab.research.google.com/github/sagagugit/Cold-Spot-Scanner/blob/main/Cold_Spot_Scanner.ipynb
- *Operating systems* Windows, Linux and Mac.
- *Programming language* Python and SQLite.
- *Other requirements* The user must be logged in with a google account. Also prefers Google Chrome or Mozilla Firefox to run the Cold Spot SCANNER.
- *License* GPL.
- *Any restrictions to use by non-academics* license needed.

### Supplementary Information

**Additional file 1**. Example calculation and output results.

## Data Availability

The data analyzed and the scripts used for the analysis during the current study are available in the Cold-Spot-Scanner repository (https://github.com/sagagugit/Cold-Spot-Scanner). The coordinates of the complexes used as examples in this paper are: https://www.rcsb.org/structure/2WPT, https://www.rcsb.org/structure/2R9P, and https://www.rcsb.org/structure/3U7Y).

## Abbreviations

PPI: Protein–protein interactions
NMR: Nuclear Magnetic Resonance
DBSCAN: Density-based spatial clustering of applications with noise
