# Supplementary material for: Cold Spot SCANNER: Colab Notebook for predicting cold spots in protein–protein interfaces
Source: BMC Bioinformatics. 2024 Apr 30;25:172. doi: 10.1186/s12859-024-05796-5 (PMC11061940; doi:10.1186/s12859-024-05796-5)
Supplement: Supplementary file 1 — Additional file 1. Example calculation and output results. [file 12859_2024_5796_MOESM1_ESM.docx]

**An example of running Cold Spot SCANNER**

#### Here, we present an example of identifying cold spots in the PDB file 1PXV, corresponding to a complex between staphostatin and staphopain.

#### First, we downloaded the file from PDB and specified the two chains that define the binding interface:


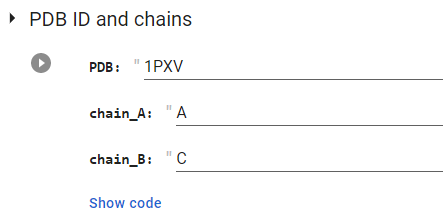


- Next, we selected the "Runtime" and “Run all” options from the menu bar in the Colab Notebook. If some calculations were run before, the user has to select "Restart and run all" to delete unwanted files from previous runs and execute the new complex.


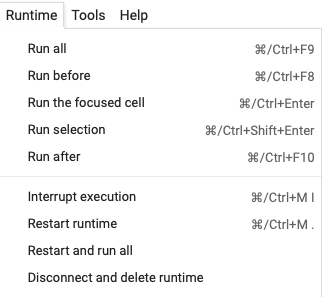


It is also possible to upload a PDB file from your computer. In this case, the user has to
remove the comment symbols (#) from all lines in the section labeled "Uploading the complex instead of PDB ID". Once the code has been uncommented, it will appear as follows:


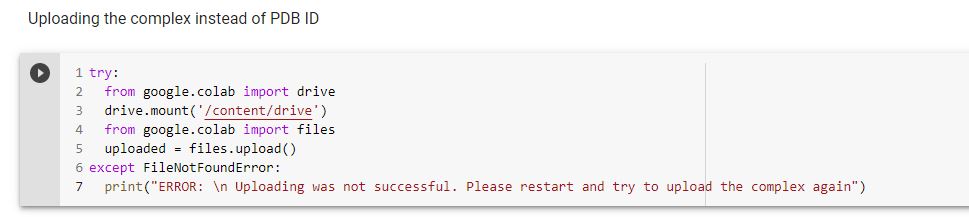


Before executing the program, the user has to specify **the same name and the same chain identifies as uploaded in the section** "PDB ID and chains". It is important that the file name has a four-letter format, similar to that of a PDB ID.


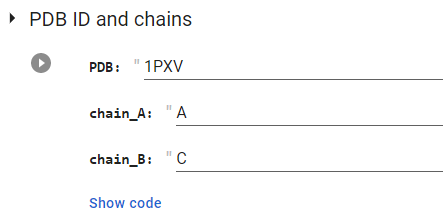


To upload your protein complex, it is necessary to authorize the notebook to access your Google Drive. Once connected to your Google Drive, you may proceed to upload the complex by selecting "Choose Files" and browsing for the desired location of the complex on your computer, followed by selecting the file.


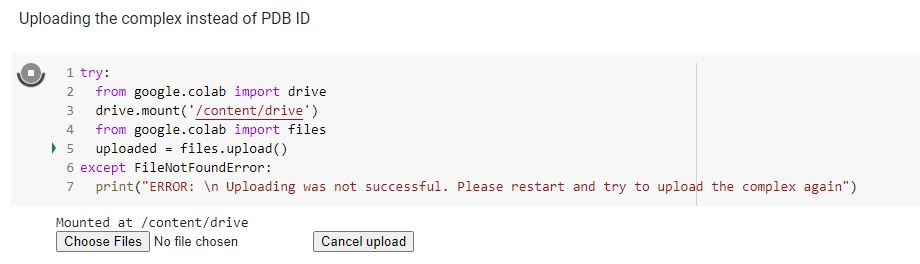


After uploading the PDB file, the program will proceed automatically, complete the running process, and generate the output.

**Visualization of the Results.**

The user can visualize cold spots directly from the Colab notebook, in the section “Visualize cold spots” as shown below. Here, the cavities are shown as red spheres, charge-hydrophobic interactions are shown in yellow, and same-charge interactions are shown in blue.


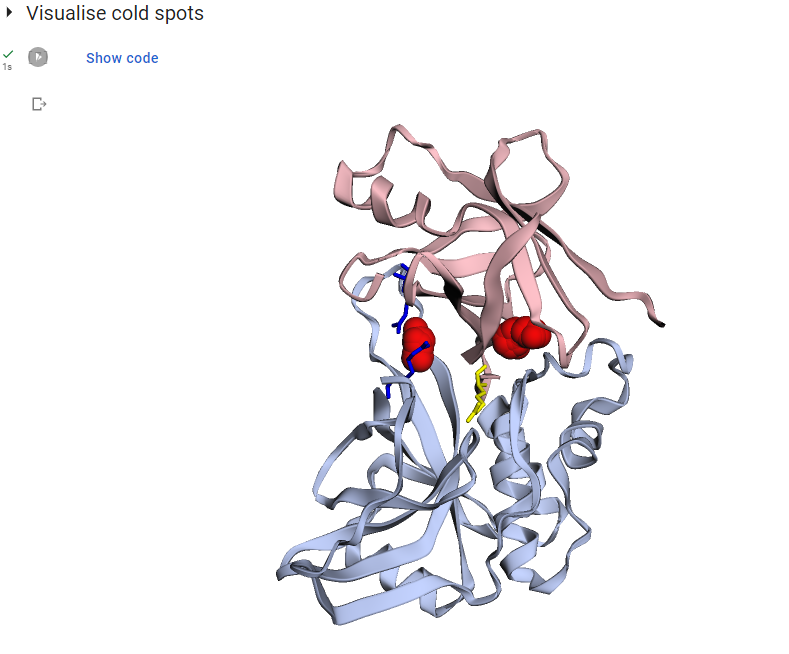


The run will also generate one zip file that contains three files for downloading including a pdb file named “Cavities_with_the_complex.pdb”, which consist of the 3D coordinates of the protein complex with identified cavities and a “PyMOL_Representation.pml”, which contain a list of PyMOL commands that will be executed to show all identified cold spots colored by type as below.


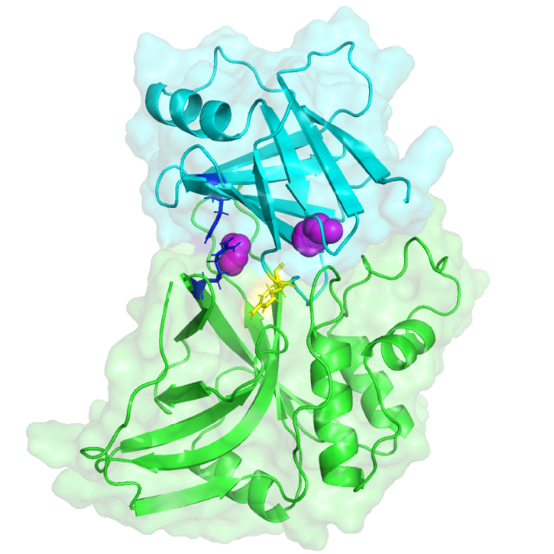


The zip file also includes a CSV file named "Identified_cold_spots.csv" containing a summary of cold spots due to each scenario:

**
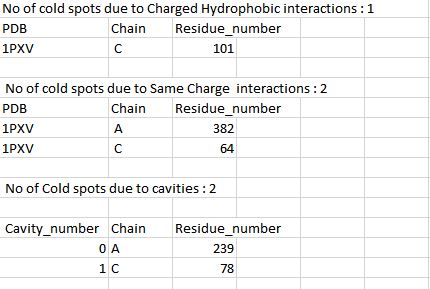
**
